# Burnout, Psychological Symptoms, and Secondary Traumatic Stress Among Midwives Working on Perinatal Wards: A Cross-Cultural Study Between Japan and Switzerland

**DOI:** 10.3389/fpsyt.2018.00387

**Published:** 2018-09-04

**Authors:** Misari Oe, Tetsuya Ishida, Céline Favrod, Chantal Martin-Soelch, Antje Horsch

**Affiliations:** ^1^Department of Neuropsychiatry, Kurume University School of Medicine, Kurume, Japan; ^2^Department Woman-Mother-Child, Lausanne University Hospital, Lausanne, Switzerland; ^3^Unit of Clinical and Health Psychology, Department of Psychology, University of Fribourg, Fribourg, Switzerland; ^4^Institute of Higher Education and Research in Healthcare, University of Lausanne and Lausanne University Hospital, Lausanne, Switzerland

**Keywords:** burnout, secondary traumatic stress, midwives, cross-cultural study, depression, anxiety

## Abstract

The aim of this study was to explore cross-cultural differences in symptoms of burnout, anxiety, depression, general psychological distress, and secondary traumatic stress between Asian (Japan) and European (Switzerland) midwives. One hundred seventy midwives participated in the study. There were significant differences in age group [χ^2^(3) = 24.2, *p* < 0.01], marital or relationship status [χ^2^(2) = 28.4, *p* < 0.01], and years of experience [χ^2^(2) = 17.8, *p* < 0.01] between the two countries. The Japanese staff were younger, more often unmarried, and had less experience than the Swiss staff. The mean score of depersonalization was significantly higher in Switzerland (4.8 ± 3.8) than in Japan (3.2 ± 3.7; |*z*| = 2.71, *p* < 0.01). The mean score of general psychological distress in the Swiss sample (12.8 ± 6.5) was significantly higher than that in the Japanese sample (10.3 ± 6.2; |*z*| = 2.04, *p* = 0.04). In addition, the mean score of secondary traumatic stress was higher in the Swiss sample (31.8 ± 9.7) than in the Japanese sample (24.1 ± 8.6; |*z*| = 4.56, *p* < 0.01). These results may reflect cultural differences such as working conditions or family environment between Japan and Switzerland.

## Introduction

The present study aimed to explore cross-cultural differences in symptoms of burnout, anxiety, depression, general psychological distress, and secondary traumatic stress between Asian (Japan) and European (Switzerland) midwives. Changes in obstetric practices, such as an increase in the proportion of childbearing women that are defined as high risk (1), may impact the well-being of midwives who face difficulties with patients at highly specialized wards or neonatal intensive care units. In particular, staff in intensive care units have been described as vulnerable compared with other healthcare professionals (2, 3). Burnout is a psychological syndrome with increased feelings of emotional exhaustion (EE), development of depersonalization (DP); i.e., negative, cynical attitudes and feelings about one's patients; and reduced personal accomplishment (PA) (4). The potential risk factors for burnout are years in the profession, workload, working long hours, shift work, demanding patient relations, lack of occupational autonomy, and work environment (1, 5–8). Studies in different countries have reported high levels of burnout among midwives, varying from 19.1% in Norway to 65% in Australia (1, 5, 7–12). Despite this strong variation in the prevalence of burnout between countries, there are to our knowledge no cross-national studies on burnout among midwives at present. Our research interest was whether there are cross-cultural differences in job-related psychological burden, including burnout, between Eastern and Western countries, because it has been reported that the levels of burnout in Japan are higher than Western countries (see below). A large-scale study examining the relationship between nurse burnout and ratings of quality of care in 53,846 nurses from six countries (USA, Canada, UK, Germany, New Zealand, and Japan) showed that nurses in Japan had the highest mean scores on EE and DP, and the lowest mean scores on PA, whereas nurses from Germany had the lowest burnout levels based on all three MBI subscales (13). This study showed that higher levels of burnout were associated with lower ratings of quality of care independently of nurses' ratings of practice environments across the countries. Although the authors of this study did not mention the reasons for the differences among the six countries, the sociodemographic data showed that the Japanese nurses were the youngest, had less nursing experience, had worked in the unit for a shorter period of time, and had the highest percentage of full time work (13). However, it remains unclear whether these findings of higher burnout scores and socio-demographic specificities will also be found in Japanese midwives. If the relationship between burnout and socio-demographic specificities was to be found in the present study, it might be useful to improve the quality of care in obstetric practices.

Psychological distress, such as anxiety and depression, is often linked to the burden of work (14). A recent review of work-related risk factors revealed that an imbalanced job design, occupational uncertainty, and lack of value and respect at the workplace explained how work may contribute to the development of depression and/or anxiety (14). In a study of Australian midwives, 17.3% reported depression, 20.4% reported anxiety, and burnout was positively correlated with depression and anxiety (10). Another study conducted in New Zealand that examined anxiety and depression showed higher levels of anxiety symptoms in employed midwives than self-employed midwives (6). So far, depression and anxiety have not been investigated in Japanese midwives.

Secondary traumatic stress is a syndrome including intrusion, avoidance, and arousal resulting from indirect traumatic exposure in a professional context (e.g., caring for traumatized patients) (15). According to a meta-analysis, the presence of secondary traumatic stress was reported in forensic nurses, emergency department nurses, oncology nurses, pediatric nurses and hospice nurses in the United States with a reported prevalence of between 25 and 38% (16). Potential risk factors were the frequency of exposure to traumatic events, history of traumatic experiences, empathy with traumatized patients, heavy workloads, increased contact with patients and long work hours (17). About one-third of labor and delivery nurses reported moderate to severe levels of secondary traumatic stress (18). Additionally, using a qualitative approach the authors performed content analysis of descriptions of experiences at traumatic childbirths and found six themes; magnifying the exposure to traumatic births, struggling to maintain a professional role while with traumatized patients, agonizing over what should have been, mitigating the aftermath of exposure to traumatic births, haunted by secondary traumatic stress symptoms, and considering foregoing carriers in labor and delivery to survive (18).

The aim of this study was to explore cross-cultural differences in symptoms of burnout, anxiety, depression, general psychological distress, and secondary traumatic stress between Asian (Japan) and European (Switzerland) midwives. Although these two countries are classed as developed countries, working conditions, and psychosocial work factors are different. Previous studies on the working conditions of nurses in Japan reported that the main differences between Japan and Western countries concern taking leave and working overtime. For Japanese workers, it is often difficult to obtain sick leave, and it is uncommon to take leave to care for sick family members, even though relevant laws exist (19). In contrast, in Switzerland employees are often offered positions based on certain percentages of work time (e.g., a job offered at 100% means full-time, up to 45–50 h a week depending on the job sector) and sick leave is allowed under the law. Regarding paid leave, the length of paid leave in Japan is between 10 and 20 working days depending on the years of continuous employment, and is therefore shorter than that in Switzerland, where it is a minimum of 4 weeks. According to a large-scale study of Japanese midwives (9), only 8.6 % of the midwives took full-length or almost full-length paid annual leave in the course of a year. With regards to overtime, a survey of nurses in the European Union indicated that 27% had worked overtime (i.e., more than their contracted hours) on their last shift (20). In Japan, a survey by the Japanese Nursing Association in 2008 showed that one out of 23 persons work at a level considered to lead to death due to overwork (defined as shifts with overtime of more than 60 h per month) (Nursing in Japan, available from https://www.nurse.or.jp/jna/english/nursing/index.html).

Taken together, these studies suggest that the working conditions in Japan are less flexible compared with European countries. Therefore, we hypothesized that the psychological burden, including burnout, anxiety, depression, general psychological distress as well as secondary traumatic stress, of midwives and nurses in Japan would be greater than that of their counterparts in Switzerland.

## Materials and methods

### Participants

Midwives working on perinatal wards and in neonatal intensive care units (NICU) at four university hospitals were recruited. Two of the convenience (i.e., not randomized) samples were in Japan (pseudonym: university hospitals A and B), and two in Switzerland (university hospitals C and D). In Japan, the questionnaires were sent by post and the completed questionnaires were anonymously returned by mail. The data were collected in February 2015 at hospital A, and of the 82 eligible staff 57 participated. The survey was conducted in February 2016 at hospital B, and of the 52 eligible staff 34 participated. Among Japanese participants (38 nurses, 51 midwives, 2 missing data), we used data of midwives (22 midwives from hospital A, and 29 midwives from hospital B) for this study. The comparison of the psychological burden between nurses and midwives is shown in Supplementary Materials. In Switzerland, an online survey was accessible during 4 months and all eligible staff received one reminder e-mail 1 month before it closed. One-hundred-and-nineteen midwives (of 209 eligible midwives) participated in Switzerland. The Swiss data reported here are part of a larger study investigating work-related well-being and mental health in midwives and NICU nurses [(21); Jacobs et al., under review]. Among sociodemographic characteristics, the Japanese participants were asked about their marital status, and the Swiss participants were asked about their relationship status. This difference is due to cultural backgrounds and we arranged the question to read naturally for each country.

### Measures

#### Burnout symptoms

Burnout symptoms were measured using the Maslach Burnout Inventory (MBI) (22). The MBI is a 22-item self-report questionnaire. Each item is rated on a Likert scale from 0 “never” to 6 “always.” All items are organized into three subscales; emotional exhaustion (9 items; MBI-EE), depersonalization (5 items; MBI-DP) and personal accomplishment (8 items; MBI-PA). The Japanese version of the MBI in this study was developed by Higashiguchi et al. (23), and showed moderate to high Cronbach's alpha coefficients; in this study, alpha coefficients were calculated after factor analysis (alpha = 0.87–0.90 for frequency of the subscales, and 0.79–0.83 for intensity of the subscales). The French version was validated by Dion & Tessier, and showed satisfactory psychometric properties (alpha = 0.90, 0.71, and 0.79, respectively for EE, PA, and DP) (24, 25). In our study, Cronbach's alpha coefficients were: 0.85 in Japan and 0.85 in Switzerland for MBI-EE; 0.76 in Japan and 0.67 in Switzerland for MBI-DP; 0.83 in Japan and 0.66 in Switzerland for MBI-PA.

#### Psychological distress

The Hospital Anxiety and Depression Scale (HADS) (26) was used to assess general psychological distress. The HADS is a 14-item self-report questionnaire measuring state anxiety (7 items; HADS-A) and depression (7 items; HADS-D). Each item is calculated from 0 to 3, with higher scores indicating higher anxiety and/or depression. Cronbach's alpha for HADS-A varied from 0.68 to 0.93 (mean 0.83) and for HADS-D from 0.67 to 0.90 (mean 0.82) (27). The Japanese version of the HADS was validated by Higashi et al. (28) and Hatta et al. (29). The French version of the HADS was developed by Lepine et al. (30) and validated by Razavi et al. (31). In this study, Cronbach's alpha values for HADS-A were 0.93 in Japan and 0.76 in Switzerland, and for HADS-D were 0.78 in Japan and 0.71 in Switzerland. These values indicate a good internal consistency of both versions of the HADS.

#### Secondary traumatic stress symptoms

Secondary traumatic stress symptoms were measured using the Secondary Traumatic Stress Scale (STSS) (32). The STSS is a 17-item self-report questionnaire with a Likert scale of 5 points from 1 “never” to 5 “very often.” All items are organized into three subscales; intrusion (STSS-I), avoidance (STSS-A), and arousal (neurovegetative activation; STSS-NA). The original version of the STSS indicated very good internal consistency and reliability with coefficient alpha levels of 0.93 for the total STSS scale, 0.80 for the intrusion subscale, 0.87 for the avoidance subscale, and 0.83 for the arousal subscale (32). The French version of the STSS was recently validated [(21); Jacobs et al., under review]. The Japanese version of the STSS has not been validated, therefore we used a back-translated version with the permission of the original author (Dr. Bride). In this study, Cronbach's alpha values for STSS-I were 0.82 in Japan and 0.80 in Switzerland; for STSS-A they were 0.84 in Japan and 0.73 in Switzerland; for STSS-NA they were 0.86 in Japan and 0.77 in Switzerland.

### Statistical analysis

All analyses were performed using IBM SPSS Statistics version 21. In this study, bootstrapping (1,000 times) was used to estimate the 95% confidence intervals. Chi-Square test was used for testing relationships between categorical variables (country, age, marital status (in Japan) or relationship status (in Switzerland), and years of experience). The Shapiro-Wilk test was used to check normal distributions of the variables. In the Japanese sample, only the MBI-EE and HADS-A showed normal distribution. In the Swiss sample, only the MBI-EE and MBI-PA showed normal distribution. According to these results, we decided to use the non-parametric analyses. Mann–Whitney test was used for independent samples to test our hypotheses of a difference in burnout, anxiety, depression, general psychological distress, and secondary traumatic stress between the two countries. We used the Kruskal–Wallis and Steel–Dwass tests to compare the differences in the subscales of the HADS, STSS, and MBI among age categories and marital status (in Japan) or relationship status (in Switzerland) in the Japanese and Swiss samples independently. All tests were two-sided and based on a 0.05 level of significance.

### Ethical considerations

This study was approved by the Ethical Committee of Kurume University, Japan (study number 14175) and the ethics committee of the Canton de Vaud, Switzerland (study number 237/2013).

## Results

### Sociodemographic characteristics

Sociodemographic characteristics are presented in Table 1. All participants were female in Japan; in Switzerland 3 (2.5%) were male (3 missing). There were significant differences in age group [χ^2^(3) = 24.2, *p* < 0.01], marital or relationship status [χ^2^(2) = 28.4, *p* < 0.01], and years of experience [χ^2^(2) = 17.8, *p* < 0.01] between the two countries. For age group, residual analysis showed that significantly more staff in Switzerland were categorized into the “40 or more” age-group (45.2%) than those in Japan (17.7%). In contrast, significantly more staff in Japan were categorized into the “18–25” age group (19.1%) and the “26–30” age group (31.4%) than those in Switzerland (2.6 and 16.5%, respectively). Therefore, the Swiss sample was significantly older than the Japanese sample. For marital status or relationship status, significantly more staff in Japan were single (58.8%) than in Switzerland (19.3%). For years of experience, significantly more staff in Switzerland were categorized into the “more than 10 years” group (60.7%) than those in Japan (37.8%). In contrast, significantly more staff in Japan were categorized into the “0–5 years” group (45.1%) and the “6–10 years” group (29.4%) than those in Switzerland (25.6 and 13.7%, respectively). Therefore, the Swiss sample had more work experience than the Japanese sample.

**Table 1:** Sociodemographic characteristics of the study participants.

		**Japan (*****N*** = **51)**		**Switzerland (*****N*** = **119)**		**χ^2^**	***p***
		***N***	**%**	***N***	**%**		
Gender	Female	51	100	113	95.0	1.3	0.2
	Male	0	0.0	3	2.5		
	No answer	0	0.0	3	2.5		
Age	18–25 years old	10	19.6	3	2.5	24.2	< 0.01
	26–30 years old	16	31.4	19	16.0		
	31–39 years old	16	31.4	41	34.5		
	40 or more	9	17.6	52	43.7		
	No answer	0	0.0	4	3.4		
Marital status (in Japan) or Relationship status (in Switzerland)	Unmarried or single	30	58.8	23	19.3	28.4	< 0.01
	Married or in relationship	21	41.2	82	68.9		
	Divorced	0	0.0	14	11.8		
	No answer	0	0.0	0	0.0		
Years of experience	0–5 years	23	45.1	30	25.2	17.8	< 0.01
	6–10 years	15	29.4	16	13.4		
	More than 10 years	13	25.5	71	59.7		
	No answer	0	0.0	2	1.7		

### Cross-cultural comparisons regarding work-related psychological symptoms

The mean scores, standard deviations, and 95% confidence intervals using bootstrapping of burnout, anxiety, depression, general psychological distress as well as secondary traumatic stress are shown in Table 2. Regarding burnout, among the three subscales there were no significant differences in the mean score of the MBI-EE between the two countries. In contrast, the mean score of the MBI-DP in Switzerland was significantly higher than that in Japan (|*z*| = 2.32, *p* = 0.02). Finally, there was no significant differences in the mean score of the MBI-PA, however, there was a tendency for higher scores in Switzerland than in Japan (|*z*| = 1.74, *p* = 0.08). Because MBI-PA is a reverse scored subscale, the burnout symptoms, as measured on this subscale, tended to be lower in Switzerland. The mean total HADS score in the Swiss sample (12.8 ± 6.5) was significantly higher than that in the Japanese sample (10.3 ± 6.2; |*z*| = 2.04, *p* = 0.04). On the subscale level, there were no significant group differences in the mean HADS anxiety (|*z*| = 1.52, *p* = 0.13) and depression scores (|*z*| = 1.72, *p* = 0.09). The mean total STSS score was significantly higher in the Swiss sample (31.8 ± 9.7) compared with the Japanese sample (24.1 ± 8.6; |*z*| = 4.56, *p* < 0.01). Similarly, the means for the STSS subscale scores were significantly higher in the Swiss sample: STSS-I (|*z*| = 2.44, *p* < 0.015), STSS-A (|*z*| = 4.76, *p* < 0.01), STSS-NA (|*z*| = 4.55, *p* < 0.01).

**Table 2:** Comparison of psychological burden between Japan and Switzerland.

	**Japan**			**Switzerland**			**|*z*|**	***p***
	**Mean**	**SD**	**Bootstrap 95% CI**	**Mean**	**SD**	**Bootstrap 95% CI**		
**BURNOUT**								
MBI emotional exhaustion	20.1	9.9	17.07–23.20	20.7	8.7	19.23–22.22	0.40	0.7
MBI depersonalization	3.2	3.7	2.08–4.33	4.8	3.8	4.09–5.40	2.71	<0.01
MBI personal accomplishment	29.7	9.5	26.73–32.75	32.9	4.8	32.02–33.72	1.74	0.08
**ANXIETY AND DEPRESSION**								
HADS total	10.3	6.2	8.48–12.25	12.8	6.5	11.73–13.95	2.04	0.04
HADS anxiety	6.5	3.7	5.44–7.80	7.7	3.8	7.01–8.40	1.52	0.13
HADS depression	3.7	3.0	2.79–4.64	5.1	3.9	4.36–5.76	1.72	0.09
**SECONDARY TRAUMATIC STRESS**								
STSS total	24.1	8.6	21.55–26.93	31.8	9.7	30.18–33.80	4.56	<0.01
STSS intrusion	7.9	3.2	7.03–8.97	9.5	3.7	8.81–10.10	2.44	0.015
STSS avoidance	8.9	3.2	7.97–10.00	11.7	3.7	11.08–12.40	4.76	<0.01
STSS neurovegetative activation	7.6	3.5	6.69–8.88	10.6	3.7	9.97–11.3	4.55	<0.01

### Comparison of psychological variables by sociodemographic characteristics

Because we found differences in some sociodemographic variables between the Japanese and the Swiss samples, we compared the differences in the HADS, STSS, and MBI subscales among age categories, marital status (in Japan) or relationship status (in Switzerland), and years of experience by each country independently. In the Japanese sample, we found significant differences in MBI-PA scores according to marital status. The Mann–Whitney test revealed that the MBI-PA score of those who were unmarried (27.2 ± 9.2) was lower than that for those who were married (34.1 ± 8.7; |*z*| = 2.20, *p* = 0.03). In addition, there was a significant difference in the STSS-A scores according to age group [χ^2^(3) = 8.4, *p* = 0.04]. The Steel–Dwass test revealed that the STSS-A score of the 18–25 age group (10.3 ± 4.0) was higher than that of the 31–40 age group (7.7 ± 2.2; |*z*| = 2.65, *p* = 0.04).In the Swiss sample, we found a significant difference in STSS-I scores according to years of experience [χ^2^(2) = 6.4, *p* = 0.04]. The Steel–Dwass test revealed that the STSS-I score of the more than 10 years' experience group (8.9 ± 3.5) was significantly lower than that of the 6–10 years' experience group (10.6 ± 2.5).

## Discussion

In this cross-sectional study, we compared burnout, anxiety, depression, general psychological distress, and secondary traumatic stress of midwives working on the maternity ward or in the NICU at two Japanese and two Swiss University Hospitals. Comparison of sociodemographic characteristics showed that Swiss midwives were older, more often in a relationship, and had more work experience. Although we could not find a cross-national study of midwives, this tendency is in line with a cross-national study of nurses (13). In this study across six countries (USA, Canada, UK, Germany, New Zealand, and Japan), Japanese nurses (all wards) were also the youngest and had less experience, whilst at the same time reported the highest rate of full-time employment (95.5%). Our results suggest similar sociodemographic differences for midwives as the ones reported for nurses. These differences in sociodemographic factors may influence the psychological burden of their work. For example, a study in Japan reported that inexperienced nurses had higher odds of developing burnout symptoms (33).

However, our results did not support our hypothesis of a greater psychological burden on midwives in Japan than in Switzerland expressed by higher scores of burnout, depression, anxiety, general psychological distress and secondary traumatic stress in the Japanese sample. The burnout subscale of depersonalization, the total HADS scores and STSS scores were significantly higher in the Swiss sample than in the Japanese sample. These results may indicate a greater psychological burden on midwives in Switzerland. These results might be explained by the different certification systems of midwives in Japan and Switzerland. In Japan, a midwifery license applicant is required to have acquired certification as a nurse either prior to obtaining the midwifery license or simultaneously. In addition, the person has to have studied midwifery for longer than 1 year at a college or training school (Japan Nursing Association. Midwifery in Japan, 2015. Available from https://www.nurse.or.jp/jna/english/midwifery/pdf/mij2015.pdf). In Switzerland, midwives are not necessarily also nurses. There are two education programs for the midwifery license; one is a course of 6 semesters plus 10 months of additional modules for persons without a nursing license. Another is a course of 4 semesters for persons with a nursing license (www.hebamme.ch). Interestingly, in Japan, nurses who were allowed to work in the same role as a midwife under the direction of obstetricians on perinatal wards and in the NICU showed significantly higher burnout, anxiety, and depression symptoms than midwives (see Supplementary Materials). Another Japanese study also demonstrated higher levels of burnout in nurses compared with midwives who were working on maternity and labor wards at 20 hospitals (34). According to these results, we speculate that midwives in Japan in this study may have had higher confidence in themselves given their longer professional training; this may have been one of the reasons as to why midwives working at university hospitals in Japan may have reported lower general psychological distress (as measured by the HADS total score) than that in Switzerland. Results from Japanese studies showing that job satisfaction of midwives was significantly higher than that of nurses (35), and that higher job satisfaction was related to lower burnout among midwives (9) may support our speculation.

In terms of burnout, a significant difference was only observed in depersonalization. Maslach et al. (4), who developed the MBI, stated “For both the Emotional Exhaustion and Depersonalization subscales, higher mean scores correspond to higher degrees of experienced burnout. Because some of the component items on each subscale had low loading on the other, exists a moderate correlation between the two subscales, which is in accord with theoretical expectations that these are separate, but related, aspects of burnout” p. 194 (4). We found few studies that mentioned the differences between emotional exhaustion and depersonalization. One of these was a recent study on the structural validity of the MBI and the influence of depressive symptoms in bank employees (36). The authors found that emotional exhaustion highly correlated with depressive items; however, suicidal ideation tended to correlate more highly with depersonalization than emotional exhaustion. However, we cannot introduce any speculations about the discrepancies given this limited information.

Our results revealed that marital status in Japan influenced personal accomplishment. Mixed results were reported between marital status and burnout. In a previous study of Japanese midwives, marital status was associated with emotional exhaustion and depersonalization (unmarried midwives showed higher scores in both), but not with low personal accomplishment (9). Another study of Japanese nurses also showed that there was no association between marital status and burnout (37). However, one study of Japanese nurses showed that married nurses had lower emotional exhaustion, lower depersonalization, and higher personal accomplishment (38). A meta-analytic study examining the relationship between age and burnout among nurses revealed that marital status was a substantial moderator of age and burnout (39). The authors reported that married female nurses with high personal accomplishment may be better protected against loss of energy and enthusiasm for their profession.

As far as we know, this is the first study comparing secondary traumatic stress symptoms between Asian and European countries. Our results showed that the midwives in Switzerland reported more severe secondary traumatic stress symptoms than midwives in Japan. In addition, sociodemographic factors such as age (in the Japanese sample) and professional experience (in the Swiss sample) influenced the differences in secondary traumatic stress in our study. Older age and more education have been shown as protective factors for secondary traumatic stress in nurses (16, 40). However, in contrast, another study in Turkey reported that nurses who were over the age of 40 years were at greater risk of secondary traumatic stress (41).

This study has several limitations. First, the work environments of the target university hospitals within one country may be different. We employed only self-report scales and did not examine the work environments or conditions, such as working time, shift length, leave and holidays. If the work environments of the target hospitals differed extensively, these differences may not be cross-cultural but may reflect the particular work environments of the hospitals themselves. Second, there was a small number of participants in this study. Third, this study was a cross-sectional study; therefore, we cannot discuss causality. Fourth, we did not ask about the number of traumatic experiences that participants had experienced either at their workplace or in their home life. Fifth, we did not consider family situations, except for sociodemographic factors. Sixth, the sample sizes were different between the two countries. Seventh, the impact of sociodemographic factors might be higher than the impact of cross-cultural issues.

Despite these limitations, this study has some strengths. We were able to use the same questionnaires and most of them had already been validated in both Japanese and French. All participants were working at university hospitals; therefore, there may be fewer differences in working conditions within the countries. Further, this is the first study that has focused specifically on midwives with a cross-cultural design.

In conclusion, this study revealed that the characteristics of the psychological burden on midwives were different in Japan and Switzerland. Future international studies on nurses and midwives examining the relationship among working conditions, family structures, and psychological burdens will be encouraged to understand cross-cultural issues.

## Author contributions

MO, CF, AH, and CM-S conceived the original idea for the study. MO, CF, and AH collected the data. MO, TI, CF, and AH performed the analyses of the data. MO drafted the manuscript. TI, CF, AH, and CM-S contributed critical comments and edited the text. All of the authors approved the final version of the manuscript.

### Conflict of interest statement

The authors declare that the research was conducted in the absence of any commercial or financial relationships that could be construed as a potential conflict of interest.
