# Supplementary material for: Burnout, Psychological Symptoms, and Secondary Traumatic Stress Among Midwives Working on Perinatal Wards: A Cross-Cultural Study Between Japan and Switzerland
Source: Front Psychiatry. 2018 Sep 4;9:387. doi: 10.3389/fpsyt.2018.00387 (PMC6131602; doi:10.3389/fpsyt.2018.00387)
Supplement: Supplementary file 1 [file Table_1.DOCX]

**Supplementary Material**

Supplementary Table 1

The Sociodemographic characteristics of the nurses and midwives, who participated the study in Japan

|  |  |  | Nurses (N = 38) | |  | Midwives (N = 51) | |
| --- | --- | --- | --- | --- | --- | --- | --- |
|  |  |  | N | % |  | N | % |
| Gender | Female |  | 38 | 100 |  | 51 | 100 |
|  | Male |  | 0 | 0.0 |  | 0 | 0.0 |
|  | No answer |  | 0 | 0.0 |  | 0 | 0.0 |
|  |  |  |  |  |  |  |  |
| Age | 18–25 years old |  | 6 | 15.8 |  | 10 | 19.6 |
|  | 26–30 years old |  | 7 | 18.4 |  | 16 | 31.4 |
|  | 31–39 years old |  | 17 | 44.7 |  | 16 | 31.4 |
|  | 40 or more |  | 8 | 21.1 |  | 9 | 17.6 |
|  | No answer |  | 0 | 0.0 |  | 0 | 0.0 |
|  |  |  |  |  |  |  |  |
| Marital status | Unmarried |  | 17 | 44.7 |  | 30 | 58.8 |
|  | Married |  | 20 | 52.6 |  | 21 | 41.2 |
|  | Divorced |  | 1 | 2.6 |  | 0 | 0.0 |
|  | No answer |  | 0 | 0.0 |  | 0 | 0.0 |
|  |  |  |  |  |  |  |  |
| Years of experience | 0-5 years |  | 9 | 23.7 |  | 23 | 45.1 |
|  | 6-10 years |  | 9 | 23.7 |  | 15 | 29.4 |
|  | More than 10 years |  | 20 | 52.6 |  | 13 | 25.5 |
|  | No answer |  | 0 | 0.0 |  | 0 | 0.0 |
|  |  |  |  |  |  |  |  |

Supplementary Table 2

Comparison of psychological burden between nurses and midwives in Japan

|  |  | Nurses | | | Midwives | | |  |  |
| --- | --- | --- | --- | --- | --- | --- | --- | --- | --- |
|  |  | mean | SD | bootstrap  95% CI | mean | SD | bootstrap  95% CI | \|z\| | p |
| Burnout |  |  |  |  |  |  |  |  |  |
|  | MBI Emotional Exhaustion | 27.9 | 10.3 | 24.54-31.24 | 20.1 | 9.9 | 17.07-23.20 | 3.14 | <0.01 |
|  | MBI Depersonalization | 4.2 | 3.8 | 3.04-5.66 | 3.2 | 3.7 | 2.08-4.33 | 1.45 | 0.15 |
|  | MBI Personal Accomplishment | 22.5 | 10.5 | 19.00-25.80 | 29.7 | 9.5 | 26.73-32.75 | 2.59 | <0.01 |
| Anxiety and Depression | |  |  |  |  |  |  |  |  |
|  | HADS Anxiety | 9.3 | 3.3 | 8.20-10.46 | 6.5 | 3.7 | 5.44-7.80 | 3.04 | <0.01 |
|  | HADS Depression | 6.7 | 3.5 | 5.60-7.89 | 3.7 | 3.0 | 2.79-4.64 | 3.76 | <0.01 |
| Secondary Traumatic Stress | |  |  |  |  |  |  |  |  |
|  | STSS Intrusion | 6.9 | 2.9 | 5.89-8.17 | 7.9 | 3.2 | 7.03-8.97 | 1.71 | 0.09 |
|  | STSS Avoidance | 9.6 | 4.7 | 8.28-12.34 | 8.9 | 3.2 | 7.97-10.00 | 0.94 | 0.3 |
|  | STSS Neurovegetative Activation | 7.3 | 3.4 | 6.14-8.90 | 7.6 | 3.5 | 6.69-8.88 | 0.42 | 0.7 |
